# Targeting protein kinase CK2 in the treatment of cholangiocarcinoma

**DOI:** 10.37349/etat.2021.00055

**Published:** 2021-10-31

**Authors:** Padma-Sheela Jayaraman, Kevin Gaston

**Affiliations:** 1 Biodiscovery Institute, University of Nottingham, NG7 2UH, UK; 2 Division of Translational Medical Sciences, School of Medicine, University of Nottingham, NG7 2UH, UK; Humanitas University, Humanitas Research Hospital, Italy

**Keywords:** Cholangiocarcinoma, protein kinase CK2, casein kinase II, dose-dependent synthetic lethality, DNA damage response, apoptosis, methuosis

## Abstract

Cholangiocarcinoma (CCA) is a disease with a very poor prognosis and limited treatment options. Although targeted therapies directed towards specific mutations found in CCA are becoming available and are showing great potential, many tumors do not carry actionable mutations and, in those that do, the emergence of drug resistance is a likely consequence of treatment. Therapeutic targeting of enzymes and other proteins that show elevated activity in CCA cells but which are not altered by mutation is a potential strategy for the treatment of target negative and drug-resistant disease. Protein kinase CK2 (CK2) is a ubiquitously expressed kinase that has increased expression and increased activity in a variety of cancer types including CCA. Several potent CK2 inhibitors are in pre-clinical development or under assessment in a variety of clinical trials often in combination with drugs that induce DNA damage. This review outlines the importance of CK2 in CCA and assesses the progress that has been made in the evaluation of CK2 inhibition as a treatment strategy in this disease. Targeting CK2 based on the expression levels or activity of this protein and/or in combination with drugs that induce DNA damage or inhibit cell cycle progression, could be a viable option for tumors that lack actionable mutations, or for tumors that develop resistance to targeted treatments.

## Cholangiocarcinoma

Cholangiocarcinoma (CCA) accounts for around 15% of primary liver cancers and this disease has both increasing incidence and increasing mortality (reviewed in [1]). In most geographical regions, CCA is a relatively rare cancer with an incidence of less than 6/100,000 of the population [1]. However, in Southeast Asia, the incidence of this disease is much higher due to widespread liver fluke infection and *Opisthorchis viverrine* and *Clonorchis sinensis* are recognized causative agents of this disease [2]. In Thailand alone, liver fluke-induced CCA results in around 20,000 deaths per year and in the wider region, this disease is a major challenge to health care systems and a significant barrier to economic development [3, 4]. Additional risk factors for CCA include cholestasis (loss of bile flow), exposure to dietary toxins and alcohol consumption, chronic hepatitis B or hepatitis C infection, and autoimmune-related diseases such as primary sclerosing cholangitis [1]. In all regions, CCA is usually advanced at presentation and the prognosis for newly diagnosed patients is often bleak. In general, only 30% of newly diagnosed patients are suitable for surgical resection and, for patients who undergo surgery, five-year survival is only around 50% [5, 6]. For patients with the non-resectable disease, the prognosis is even worse with overall survival of only around 1 year [7]. In Southeast Asia, the prognosis for newly diagnoses patients is dire. In Thailand, for example, only around 2% of patients with intrahepatic CCA are eligible for surgical resection and the five-year survival for these patients is only around 20% while for non-resectable disease the median overall survival is just 2 months [3]. The rising incidence of this disease and the very poor outlook for patients make this a global health problem that requires new methods for earlier diagnosis as well as new treatment approaches.

CCA can be divided into distinct disease types based on anatomical location; intrahepatic CCA is located in the bile ductules and represents about 15% of cases, while extrahepatic CCA comprises perihilar CCA, located in the left and right hepatic ducts as they join and emerge from the liver and representing about 55% of cases, and distal CCA, located in the common bile duct and representing around 30% of cases [1]. Genomic sequencing has shown that intrahepatic CCA often displays an increased frequency of mutations in genes encoding isocitrate dehydrogenase (*IDH*, *IDH1*, *IDH2*) and Kirsten rat sarcoma 2 viral oncogene homolog (*KRAS*), fibroblast growth factor receptor (*FGFR*) *2* gene fusion events, and tumour protein p53 (*TP53*) mutations whereas extrahepatic CCA often shows an increased frequency of mutations in *KRAS* and *TP53* [8, 9]. In addition, liver fluke-associated CCA and non-liver fluke-associated CCA appear to have different mutational profiles; liver fluke-associated CCA displays a higher frequency of erb-b2 receptor tyrosine kinase 2 (*ERBB2*) gene amplification and *TP53*, and SMAD family member 4 (*SMAD4*) mutations, and fluke-negative CCA display programmed cell death 1 ligand 1 (PD-L1)/programmed cell death 1 ligand 2 (PD-L2) protein expression, mutations in *IDH1/2* and breast-cancer susceptibility gene 1-associated protein 1 (*BAP1*), and *FGFR*-related gene rearrangements [8, 10, 11]. These differences in mutational profiles may be related to the different aetiology of these disease states including the immune response and the relative importance of different disease co-factors such as dietary components [1].

CCA is characterized by a tumor stroma that promotes aggressive tumor behavior through paracrine signaling and this offers opportunities for new drug treatments [12–14]. Cancer-associated fibroblasts (CAFs) derived from local fibroblasts (hepatic stellate cells, HSCs) or bone marrow-derived mesenchymal stem cells(MSCs) are stromalcells that become activated inresponseto signalsfrom CCAcells and through interaction with other stroma cells [15, 16]. Tumor-associated neutrophils and macrophages are associated with poor prognosis in a variety of cancer types [17] and a risk signature based on the presence of tumor-associated neutrophils and FOXP3^+^ regulatory T cells (T_regs_) in extrahepatic CCA has been identified [18]. CD4^+^ T_regs_ are also associated with poor prognosis and can suppress host immune responses and promote tumor growth and invasion [19]. Multiple other cell types in the tumor stroma, including blood platelets, T helper cells [Th, responsible for immune surveillance such as interleukin-17 (IL17)-producing T cells (Th17 cells) associated with autoimmunity], and T_regs_ (that inhibit the activity of Th cells) produce transforming growth factor (TGF) β and other factors that act on the CCA cells [20]. TGFβ also acts on the immune cells favoring the development of T_regs_ and enabling the evasion of immune responses by, for example, prostate cancer cells [21].

## Current treatments

Current treatment options for CCA have been recently reviewed [1, 5, 22] and here we will only briefly summarise this topic. Surgical resection of CCA offers the potential of curative treatment. This can be followed by adjuvant chemotherapy with capecitabine, a pro-drug which following conversion to 5-fluorouracil, blocks DNA replication by acting as an inhibitor of thymidylate synthase [23]. Liver transplantation is also potentially curative for intrahepatic and perihilar CCA but this is not widely available or likely to become so in the near future. For non-resectable diseases, the first-line treatment is cisplatin and gemcitabine [7, 22, 24]. Cisplatin induces DNA damage in the form of intrastrand diadducts, and interstrand cross-links which can block DNA replication and initiate cell death via apoptosis. Similarly, gemcitabine inhibits DNA replication and induces apoptosis. Following disease progression, 5-fluorouracil/leucovorin/oxaliplatin (FOLFOX) is a second-line treatment [25]. Leucovorin increases inhibition of thymidylate synthase by 5-fluorouracil resulting in more effective inhibition of DNA replication while oxaliplatin induces DNA damage and blocks DNA replication. None of these treatments specifically target cancer cells and in all cases, drug-resistant cells can emerge through, for example, the up-regulation of DNA damage repair processes [1]. Moreover, 5-year survival is only around 50% for resectable disease and the prognosis for the non-resectable disease is much worse [5–7]. For these reasons, new treatments modalities are urgently needed.

## New treatment opportunities

Immunotherapy has shown promise in the treatment of CCA [26]. Blocking the interaction between the PD-1 protein expressed on T cells, and the PD-L1, PD-L2 proteins expressed on cancer cells can result in the activation of intrinsic T cell cytotoxicity and cancer cell killing. CCA cells commonly express PD-L1 and PD-1 expressing lymphocytes infiltrate CCA tumors [27, 28]. PD-L2 expression is also seen in some CCA cells. The anti-PD-1 antibody pembrolizumab has shown promising results in the treatment of CCA [29, 30]. However, early data indicates that only around 40% of patients might respond positively to this treatment. This approach will need to be improved if it is to benefit the majority of patients and this could be done by new immunotherapy-drug combinations and/or new immunotherapies that restore immune surveillance [31, 32].

Heparin-binding growth factors (HB-GFs), including vascular endothelial growth factors (VEGFs) and platelet-derived growth factors (PDGFs) released from stromal cells, support tumor growth and progression to a more aggressive, invasive phenotype and CCA cells produce and respond differently to HB-GFs through aberrant receptor/co-receptor expression/activity [12]. However, the inhibition of angiogenesis using bevacizumab to target VEGF-A, or sorafenib to block VEGF receptor activity, has not proven to be a highly effective treatment for CCA in clinical trials [33, 34] and lymphangiogenesis inhibition, using PDGF receptor (PDGFR) inhibitors or VEGF-C/VEGF receptor (VEGFR) 3 inhibitors has promise but has not yet been tested in clinical trials [35]. Blood platelets are commonly found in tumors and act as a source of PDGF and other growth factors including TGFβ. PDGF has been shown to increase CCA cell invasion [36] and to increase VEGF-C and VEGF-A production by tumor-associated fibroblasts [35]. Blocking the activity of PDGF and other growth factors released by blood platelets could also be of value in the treatment of CCA and further work is needed in this area.

Several recent studies have characterized the mutational landscape in CCA cells and identified mutations that can be targeted and these approaches have recently been reviewed in detail [1]. For example, sequencing of tumor DNA can identify targetable mutations such as gene fusion events that involve the *FGFR2* gene [37, 38]. *FGFR2* fusions are found in around 10% of CCA can be targeted by kinase inhibitors such as pemigatinib and infigratinib [39]. In 2020, the United States Food and Drug Administration granted approval for the use of pemigatinib in the treatment of CCA patients with *FGFR2* gene fusions. However, many if not most CCA tumors lack mutations that are currently targetable, and moreover, resistance to targeted therapies are quick to emerge. One possible approach in these cases is to target enzymes and other proteins that are expressed at higher levels in tumor cells than in normal cells. This is known as dose-dependent synthetic lethality. For example, aberrant expression of the proline rich homeodomain protein (PRH)/haematopoietically expressed homeobox (HHEX) transcription factor in CCA cells results in increased cyclin-dependent kinase (CDK) 4/6 activity and a consequent increase in sensitivity to the CDK4/6 inhibitor palbociclib [40]. This approach can also be used against targets such as growth factors that are functional in the tumor microenvironment rather than in the tumor cells.

## Protein kinase CK2

Protein kinase CK2 (CK2, formerly known as casein kinase 2) is a serine/threonine kinase that phosphorylates a range of intracellular and extracellular target proteins that are important in many biological processes including cell proliferation, cell migration, and invasion, all key elements of the cancer cell phenotype. Excellent comprehensive reviews of CK2 structure and function are available [41–43] and here we will only briefly introduce CK2 before reviewing its relevance in CCA and cellular processes relevant to this disease and its treatment.

CK2 is a hetero-tetrameric protein with two catalytic α subunits and two regulatory β subunits. There are two highly related CK2 α isoenzymes encoded by the *CSNK2A1* and *CSNK2A2* genes while there is a single form of the regulatory β subunit, encoded by the *CSNK2B* gene. CK2 is a constitutively active protein kinase that transfers a phosphate from adenosine triphosphate (ATP) or guanosine triphosphate (GTP) to serine or threonine residues in target proteins in sites corresponding to the consensus sequence S/T–X–X–D/E where X can be any amino acid or S/T–X–X–pS where pS represents phosphoserine [44, 45]. The later sequence allows the regulation of phosphorylation by CK2 by other kinases. In some cases, CK2 can phosphorylate tyrosine residues although this is a much rarer event. Some CK2 target proteins bind to the regulatory CK2 β subunit and this can increase or decrease target phosphorylation by the CK2 α subunits [46]. Many signaling pathways alter CK2 activity through either (i) direct effects on CK2 enzymatic activity, (ii) the creation of sites for CK2 phosphorylation by other phosphorylation events, (iii) transcriptional regulation of the genes encoding CK2 subunits, (iv) the alteration of CK2 intracellular localization, (v) changes in regulatory interactions with the β subunit, or possibly though (vi) β subunit phosphorylation [42]. Increased CK2 activity results in the up-regulation of cell proliferation via multiple mechanisms including, for example, increased phosphorylation of the PRH/HHEX transcription factor in vascular smooth muscle cells [47].

## CK2 and cell signaling pathways

CK2 is important in multiple signal transduction pathways and many of these pathways regulate CK2 activity (reviewed in [42]). This creates regulatory feedback loops many of which are poorly understood. A full description of the interactions between these signaling pathways and CK2 is beyond the scope of this review. However, several growth factor receptor tyrosine kinases including PDGFR and epidermal growth factor receptor (EGFR), activate Src family tyrosine kinases which in turn activate mitogen-activated protein kinase (MAPK) and other kinases important in signal transduction including CK2. For example, Lyn and c-Fgr are Src family tyrosine kinases that up-regulate CK2 activity through the phosphorylation of CK2 α subunits [48]. The inhibition of Src activity using drugs such as dasatinib results in the indirect inhibition of CK2 [49, 50]. CK2 is also important in inflammatory responses and other signaling pathways that involve nuclear factor-kappa B (NF-κB) since CK2 phosphorylates inhibitor of NF-κB (IκB) triggering NF-κB activation [51].

## CK2 in DNA repair

CK2 is important in many aspects of the cellular response to DNA damage and the consequent DNA repair processes. Several proteins important in the DNA damage response or DNA repair are CK2 substrates. The p53 tumor suppressor protein (TP53) for example can induce cell cycle arrest or apoptosis in response to DNA damage and phosphorylation of p53 by CK2 required for p53 function [52–54]. However, CK2 can also interact with substrate proteins and non-substrate proteins involved in DNA repair possibly in order to recruit CK2 to specific sites. p53 is a case in point since p53 interacts with the CK2 regulatory subunit as well as being a CK2 substrate [53, 55]. A recent comprehensive review of these interactions and the role of CK2 in DNA damage and DNA repair is available [56] and here we will focus on the importance of CK2 in the repair of DNA damage induced by chemotherapeutic drugs such as cisplatin.

DNA damage results in the formation of DNA repair foci characterized by the accumulation of phosphorylated histone variant H2AX (γH2AX) which in turn recruits the Mre11-Rad50-Nbs1 (MRN) complex to initiate repair. Ataxia-telangiectasia mutated (ATM) kinase is primarily responsible for the phosphorylation of H2AX in response to DNA damage [57]. However, CK2 colocalizes with H2AX in DNA repair foci, and the inhibition of CK2 delays the clearance of these foci suggesting that CK2 activity is required for the completion of DNA repair and/or foci disassembly [58]. Cisplatin-induced DNA damage recruits x-ray repair cross-complementing 1 (XRCC1) which in turn recruits multiple DNA repair proteins. XRCC1 is a CK2 substrate and CK2-dependent phosphorylation is required for the incorporation of phosphorylated XRCC1 into DNA repair foci [59]. Inhibition of CK2 reduces the phosphorylation of XRCC1 in response to cisplatin treatment resulting in increased apoptotic cell death in ovarian carcinoma cell lines and reduced tumor growth in mouse xenograft cancer models [60]. Similar results were seen when combining CK2 inhibition with gemcitabine treatment which also induces phosphorylation of XRCC1 [60]. The combination of CK2 inhibition with cisplatin or gemcitabine also increased the levels of γH2AX in these cells [60].

## CK2 expression and activity in cancer cells

**Figure 1.**
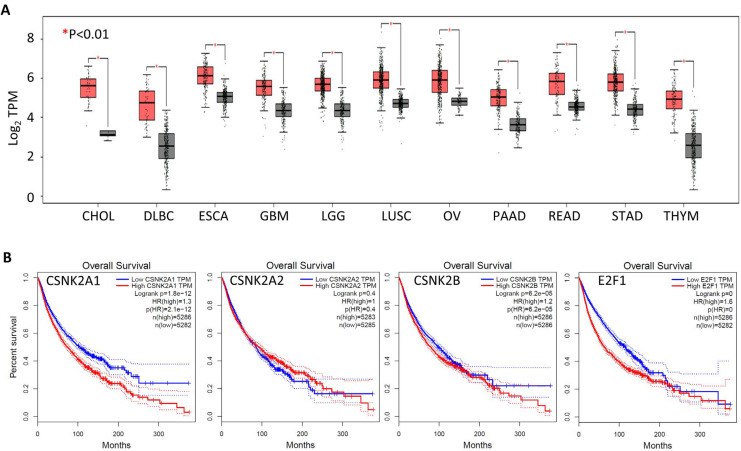
High *CSNK2A*1 mRNA expression is associated with reduced overall survival across cancer types. (A) Box plot of *CSNK2A1* mRNA expression levels in multiple cancer types. In each case tumor samples are in red and normal samples are in black. Only tumor types with a statistically significant difference in expression between cancer and control samples (*P* < 0.01) are shown. TPM: transcripts per million; CHOL: cholangiocarcinoma; DLBC: diffuse large B-cell lymphoma; ESCA: esophageal carcinoma; GBM: glioblastoma multiforme; LGG: lower grade glioma; LUSC: lung squamous cell carcinoma; OV: ovarian serous cystadenocarcinoma; PAAD: pancreatic adenocarcinoma; READ: rectum adenocarcinoma; STAD: stomach adenocarcinoma; THYM: thymoma; (B) Kaplan-Meier survival analysis of mRNA expression versus overall survival for *CSNK2A1*, *CSNK2A2*, and *CSNK2B* (with *E2F1* shown for comparison) across all TCGA cancer types. In each case, low expression is shown in blue and high expression is shown in red. High *CSNK2A1*, high *CSNK2B*, and high *E2F1* expression are all associated with reduced overall survival. All data were obtained using GEPIA2 from TCGA data sets. E2F1: E2F transcription factor 1; GEPIA2: gene expression profiling interactive analysis 2

The genes encoding CK2 subunits are not commonly mutated in cancer cells; *CSNK2A1*, *CSNK2A2*, and *CSNK2B* are mutated in around 2%, 1%, and 1%, respectively, of 10,967 patient samples from 32 studies in The Cancer Genome Atlas (TCGA; https://www.cancer.gov/tcga). *CSNK2A1* is amplified in some cancers albeit at a relatively low frequency. However, over-expression of CK2 α in conjunction with the Myc proto-oncogene protein can bring about acute lymphocytic leukemia in transgenic mice [61] and overexpression of CK2 α in the mammary gland can bring about hyperplasia and dysplasia [62, 63]. Moreover, *CSNK2A1* mRNA is highly expressed in several tumor types including CCA (Figure 1A), and, across all tumor types, high *CSNK2A1* expression correlates with poor overall survival (Figure 1B). CK2 enzymatic activity is increased in several tumor types including breast cancer, prostate cancer, and lung cancer [62, 64–67]. Increased CK2 activity results in increased phosphorylation of CK2 target proteins that include a variety of oncoproteins proteins and tumor suppressor proteins. Increased phosphorylation of oncoproteins by CK2 can increase their activity whilst increased phosphorylation of tumor suppressor proteins can result in their inactivation. For example, CK2 mediated phosphorylation of protein kinase B (PKB)/AKT increases its oncoprotein activity while CK2 mediated phosphorylation of the phosphatase and tensin homology protein (PTEN) and the promyelocytic leukemia protein (PML), reduces their tumor suppressor activity [68–70]. These, and other related observations, led to the suggestion that the inhibition of CK2 activity could be useful in cancer therapy as well as in numerous other disease states linked to dysregulated cell proliferation [41, 71]. Our work has shown that CK2 phosphorylation of the PRH/HHEX transcription factor prevents this protein from binding to DNA and blocks the tumor suppressor activity of this protein in leukemic cells [66, 72, 73]. However, the *PRH/HHEX* gene that encodes PRH is a member of a select group of genes known as proto-oncogenes with tumor suppressor function [74]. PRH is not expressed in normal bile duct epithelial cells but it is expressed in CCA cells and this context, it acts as an oncoprotein [40]. Further work is required to determine whether phosphorylation of PRH occurs in CCA cells and whether this down-regulates PRH activity.

## CK2 inhibitors

A variety of CK2 inhibitors have been characterized in different degrees of detail and recent comprehensive reviews are available [71, 75, 76]. Many of these inhibitors bind to the ATP/GTP binding site and act as competitive inhibitors of CK2. Although several of these competitive inhibitors including 4,5,6,7-tetrabromobenzotriazole (TBB) and 4,5,6,7-tetrabromo-*N*,*N*-dimethyl-1*H*-benzimidazol-2-amine (DMAT) are useful experimental tools, most are unsuitable for clinical use. However, CX-4945 (also known as silmitasertib) is a competitive inhibitor that is orally administered and highly specific and this drug is currently under investigation in a number of clinical trials [43, 77]. Other CK2 inhibitors act independently of the ATP/GTP pocket. For example, CIGB-300 blocks substrate phosphorylation by binding to the phosphoacceptor domain of CK2 substrates as well as inhibiting CK2 activity by binding directly to CK2 [78, 79]. CIGB-300 is also being investigated in clinical trials and appears to be safe and well-tolerated [80, 81]. Allosteric inhibitors that bind away from the active site and block CK2 activity, and bi-substrate inhibitors, that compete with ATP and bind to the phosphoacceptor substrate-binding site, are also being developed but are not yet in clinical trials [82, 83]. Perhaps most interesting are multi-target inhibitors that combine CK2 inhibitors such as CX-4945 with other drugs such as DNA damage-inducing cisplatin [84, 85].

## CK2 in CCA

The genes encoding CK2 subunits (*CSNK2A1*, *CSNK2A2*, or *CSNK2B*) are not mutated in CCA. However, the expression of these genes is increased in CCA cells compared to matched normal liver tissue, and the CK2 α, CK2 α’, and CK2 β proteins are all highly expressed in CCA cell lines [86]. Over-expression of the CK α subunits in CCA cells is consistent with the pro-tumourigenic roles of these proteins in other contexts. However, the over-expression of CK2 β is in contrast to the generally anti-tumourigenic view of this protein gained from studies of other cell types. Importantly, the inhibition of CK2 has been shown to reduce the viability of CCA cells *in vitro* [87, 88] and to reduce the growth of CCA cells in a xenograft mouse model [88]. The increased activity of CK2 seen in CCA makes this enzyme an attractive therapeutic target, especially in tumors that lack actionable mutations. Moreover, a clinical trial using CX-4945 in combination with cisplatin and gemcitabine (ClinicalTrials.gov identifier: NCT02128282) has reported promising preliminary data [89]. Here we will outline the effects of CK2 inhibition on CCA cells, alone or in combination with other drugs, and discuss the potential for this approach in the treatment of this disease.

## CK2 inhibition reduces CCA cell proliferation and cell invasion

Treatment of CCA cell lines with CX-4945 reduces the growth of the cell population over time [87]. 5-bromo-2’-deoxyuridine (BrdU) incorporation assays show reduced cell proliferation in the presence of CX-4945 [87]. Treatment with CX-4945 also increases cell death. Interestingly, CX-4945 treatment appears to induce two forms of programmed cell death, methuosis, and apoptosis, as discussed in detail below.

Treatment with 10 μmol/L CX-4945 also reduces CCA cell migration and reduces the ability of these cells to invade Matrigel [87], and extracellular matrix that models the basement membrane that surrounds epithelial tissues. This appears to be a direct consequence of CK2 inhibition by CX-4945 since the combined knockdown of CK2 α and CK2 α’ expression using small interfering RNA (siRNA) also inhibits cell invasion [87]. Interestingly, lower doses of CX-4945 appear to increase cell invasion possibly due to increased expression of matrix metallopeptidases [87]. Any stimulatory effects of low doses of CX-4945 on cell invasion could have negative consequences in clinical applications.

## CK2 inhibitors can induce apoptosis and methuosis in CCA cells

**Figure 2.**
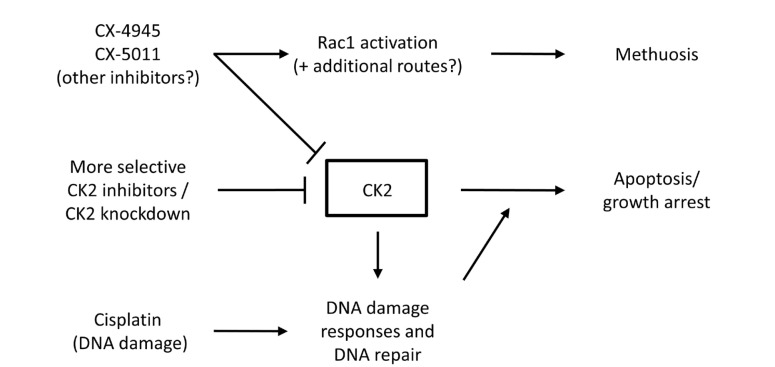
CK2 inhibitors induce cell death by multiple mechanisms. A schematic representation of the effects of CK2 inhibitors on cell fate. Methuosis is a rapid response to some CK2 inhibitors occurring within a few hours. Apoptosis is a slower response to CK2 inhibition occurring after 24 h or more. CK2 inhibition in conjunction with chemotherapeutic drugs that induce DNA damage results in increased levels of cell death and this may be useful in cancer treatment

Apoptosis is a form of programmed cell death that involves chromatin compaction, DNA fragmentation, cytoplasmic blebbing/boiling, and cell shrinkage [90]. In contrast, methuosis is a form of cell death characterized by the presence of large vacuoles that derive from macropinosomes, in the absence of cell shrinkage and chromatin compaction [91]. Treatment of CCA cells with the CK2 inhibitor CX-4945 can induce both apoptosis and methuosis. Apoptosis is induced by CX-4945 ≥ 10 μmol/L although only after relatively long treatments of 48 h or 72 h [88]. In contrast, treatment with 10 μmol/L CX-4945 can induce methuosis after just a few hours and this form of cell death is even more pronounced with higher doses [87]. This suggests that the reduction of CCA cell viability following CX-4945 treatment arises from an early effect on methuosis combined with a longer-term effect on apoptosis. Interestingly, the induction of methuosis does not appear to be due to the effects of CX-4945 on CK2 since the removal of the CK2 protein using siRNA does not prevent CX-4945-induced methuosis [87]. Moreover, although CX-4945 and the closely related drug CX-5011 induced vacuolization in HepG2 cells, other inhibitors of CK2 including TBB, failed to induce vacuolization [92]. The Rac1 GTPase plays a central role in growth factor signaling via Ras activation and other signaling pathways and is important in methuosis [91]. Recent elegant experiments have shown that the inhibition of Rac1 activity or Rac1 knockdown using siRNA, significantly reduces the effects of CX-5011 on vacuolization [92]. Moreover, CX-5011 treatment results in Rac1 activation [92]. The effects of different CK2 inhibitors on methuosis and apoptosis are summarized in Figure 2. The activation of Rac1 by these CK2 inhibitors could also be important in the effects of these drugs on cell migration and invasion. Moreover, CX-4945 and CX-5011 have other off-target effects including the alteration of mRNA splicing through the inhibition of Cdc2-like kinases [93], and these off-target effects could also be in part responsible for CX-4945/CX-5011-induced methuosis.

## Drug combinations

The combination of CX-4945 with drugs that block AKT activity or TGF β signaling results in an additive inhibitory effect on CCA cells *in vitro* [94]. Similarly, loss of CK2 α activity by CRISPR/Cas9 genome editing increases the inhibitory effects of 5-fluorouracil or gemcitabine on the viability of CCA cells [86]. As might be expected, the combination of CX-4945 with either cisplatin or gemcitabine results in increased cytotoxicity against CCA cell lines [88]. The combination of CX-4945 with these drugs has an additive inhibitory effect on cell viability (Figure 2). Importantly, the triple combination of CX-4945 with cisplatin and gemcitabine reduced the growth of CCA tumors in a xenograft mouse model [88].

The sequence of drug administration is often of importance when drugs are combined. Administration of a drug that blocks the repair of DNA damage followed by the addition of a drug that induces DNA damage might logically be expected to be more efficacious than the reverse combination. However, this is not the case since treatment of ovarian cancer cell lines with cisplatin or gemcitabine prior to CX-4945 treatment resulted in more cell death compared to the reverse sequence of drug additions [60]. This drug combination is the subject of ongoing clinical trials which propose to treat patients with CX-4945 prior to cisplatin treatment. However, treatment with cisplatin prior to CX-4945 treatment may be of greater efficacy. The use of multi-target inhibitors, such as Cx-platin which combines CX-4945 and cisplatin to inhibit CK2 and induce DNA damage [84], might also be more effective in blocking the growth of CCA tumors.

## Conclusions

The inhibition of CK2 activity could be of value in the treatment of CCA most likely in combination with drugs that induce DNA damage. CK2 expression of CK2 activity in CCA could be used to select patients with tumors that might respond well to these approaches. However, further work is required to establish the most efficacious drug combinations and identify patients who will benefit from this approach. This approach may be particularly useful for the treatment of tumors that lack actionable mutations or are resistant to targeted treatments. In the context of many countries with a high incidence of CCA, targeted therapies are unlikely to be widely available in the foreseeable future due to resource limitations. Combination therapies that make use of CK2 inhibitors may provide treatment options that are both efficacious and cost-effective. Local clinical trials are required to evaluate the potential of these approaches for the treatment of this disease.

## Abbreviations

ATP: adenosine triphosphate
CCA: cholangiocarcinoma
E2F1: E2F transcription factor 1
FGFR: fibroblast growth factor receptor
GTP: guanosine triphosphate
HHEX: haematopoietically expressed homeobox
IDH: isocitrate dehydrogenase
NF-κB: nuclear factor-kappaB
PD-1: programmed cell death 1
PDGFs: platelet-derived growth factors
PD-L1: programmed cell death 1 ligand 1
PD-L2: programmed cell death 1 ligand 2
PRH: proline rich homeodomain
siRNA: small interfering RNA
TGF: transforming growth factor
Th: T helper cells
TP53: tumor protein p53
T_regs_: regulatory T cells
VEGFs: vascular endothelial growth factors
XRCC1: x-ray repair cross-complementing 1

## Declarations

### Copyright

© The Author(s) 2021.
